# Assessing ChatGPT's Capability in Addressing Thyroid Cancer Patient Queries: A Comprehensive Mixed-Methods Evaluation

**DOI:** 10.1210/jendso/bvaf003

**Published:** 2025-01-13

**Authors:** Matthew A Gorris, Reese W Randle, Corey S Obermiller, Johnson Thomas, David Toro-Tobon, Sophie Y Dream, Oliver J Fackelmayer, T K Pandian, Sarah E Mayson

**Affiliations:** Division of Endocrinology and Metabolism, Wake Forest University School of Medicine, Winston Salem, NC 27101, USA; Department of Surgery, Section of Surgical Oncology, Wake Forest University School of Medicine, Winston Salem, NC 27101, USA; Informatics and Analytics, Department of Internal Medicine, Atrium Health Wake Forest Baptist, Winston Salem, NC 27157, USA; Department of Endocrinology, Mercy Health, Springfield, MO 65807, USA; Division of Endocrinology, Diabetes, Metabolism, and Nutrition, Mayo Clinic, Rochester, MN 55905, USA; Division of Surgery, Medical College of Wisconsin, Milwaukee, WI 53226, USA; Division of General, Endocrine and Metabolic Surgery, University of Kentucky, Lexington, KY 40508, USA; Department of Surgery, Section of Surgical Oncology, Wake Forest University School of Medicine, Winston Salem, NC 27101, USA; Division of Endocrinology, Metabolism and Diabetes, University of Colorado School of Medicine, Aurora, CO 80045, USA

**Keywords:** thyroid cancer, ChatGPT, artificial intelligence

## Abstract

**Context:**

Literature suggests patients with thyroid cancer have unmet informational needs in many aspects of care. Patients often turn to online resources for their health-related information, and generative artificial intelligence programs such as ChatGPT are an emerging and attractive resource for patients.

**Objective:**

To assess the quality of ChatGPT's responses to thyroid cancer-related questions.

**Methods:**

Four endocrinologists and 4 endocrine surgeons, all with expertise in thyroid cancer, evaluated the responses to 20 thyroid cancer-related questions. Responses were scored on a 7-point Likert scale in areas of accuracy, completeness, and overall satisfaction. Comments from the evaluators were aggregated and a qualitative analysis was performed.

**Results:**

Overall, only 57%, 56%, and 52% of the responses “agreed” or “strongly agreed” that ChatGPT's answers were accurate, complete, and satisfactory, respectively. One hundred ninety-eight free-text comments were included in the qualitative analysis. The majority of comments were critical in nature. Several themes emerged, which included overemphasis of diet and iodine intake and its role in thyroid cancer, and incomplete or inaccurate information on risks of both thyroid surgery and radioactive iodine therapy.

**Conclusion:**

Our study suggests that ChatGPT is not accurate or reliable enough at this time for unsupervised use as a patient information tool for thyroid cancer.

Differentiated thyroid cancer affects a substantial number of individuals globally, with an incidence of 821 000 cases worldwide in 2022 [1]. Data from the National Cancer Institute's Surveillance, Epidemiology, and End Results program estimate approximately 44 000 new diagnoses of thyroid cancer will be made in the United States alone in 2024 [2]. The majority of these diagnoses will be comprised of differentiated thyroid cancers (DTC), which also have the best prognosis. The combined 5-year relative survival rate in patients with DTC is 98%, and many patients experience long-term durable remission. Despite the relatively favorable prognosis, data suggest patients with DTC report lower health-related quality of life and experience significant cancer-related fear and anxiety that can impact decision-making for treatment [3-11]. One avenue to improve patient support involves enhancing access to reliable information. Published literature suggests patients with thyroid cancer have unmet informational needs in many aspects of care, including general information on thyroid cancer, details regarding treatment and surveillance, awareness of resources for emotional support including patient led groups, and managing the cost of care [12-14].

Patients frequently turn to the internet to answer their health-related questions. Between 65% and 80% of cancer patients utilize the internet to seek information [15-17]. Despite the plethora of information online, patients frequently encounter difficulties in finding *reliable* and *understandable* resources that cater to their specific needs [14]. The vast amount of medical literature, varying sources of information, and the heterogeneity of patient experiences contribute to these challenges.

Generative artificial intelligence (AI) is emerging as a useful tool in various aspects of medicine, and endocrinology is no exception [18, 19]. It can be used to create content such as text, imaging, and other media by leveraging natural language processing and deep-learning algorithms. Specifically, AI chat bots, such as ChatGPT, have become increasingly popular as a resource for the general public. These chat bots are trained on vast amounts of data and can answer questions in a conversational way. Given their availability and ease of use, patients may turn to generative AI programs to answer health-related questions. Several studies have evaluated ChatGPT's ability to answer patients’ medical questions, with mostly promising results [20-25]. Recently, 2 studies have examined ChatGPT's ability to answer patient questions on both thyroid nodules and general thyroid-related questions [26, 27]. In the first study, ChatGPT's responses to thyroid nodule questions were deemed correct 69% of the time. In the second study, ChatGPT's responses scored higher when compared to responses from 2 separate surgeons. Although these studies have begun to explore the strengths and weaknesses of ChatGPT as a tool for patient education, more research in this area is needed. The purpose of this study is to evaluate ChatGPT's responses to questions regarding thyroid cancer using a mixed-methods approach.

## Methods

This study did not involve any human subjects and therefore was exempt from institutional review board review. Twenty thyroid cancer-related questions were generated based on data from a systematic review of unmet information needs among patients with thyroid cancer [14]. These questions were organized into the following categories: disease-related information (3 questions), treatment (7 questions), aftercare (7 questions), psychosocial needs (2 questions), and alternative/complementary medicine (1 question). The questions were entered twice into ChatGPT version 3.5 on 2 separate days in February 2024; each question was entered into its own individual chat thread. This generated 2 separate sets of responses, AI1 and AI2. Both sets of responses were submitted as supplemental material and can be accessed through the Harvard Dataverse repository [28].

Four endocrinologists and 4 endocrine surgeons reviewed the ChatGPT responses. These 8 reviewers represented 6 different institutions across the United States and were selected based on their reputation as experts in thyroid cancer. The reviewers were asked to rate the responses from ChatGPT on a 7-point Likert scale by responding to the following 3 statements: (1) The response is accurate, (2) The response is complete/comprehensive, and (3) I am satisfied with the response. The Likert-scale values were as follows: 1 = strongly disagree, 2 = disagree, 3 = somewhat disagree, 4 = neutral, 5 = somewhat agree, 6 = agree, 7 = strongly agree. The reviewers were also encouraged to provide free-text comments about the responses. The reviewers were asked to rate both sets of responses, at least 1 week apart. Four of the reviewers began with AI1 and the other 4 began with AI2.

In addition to the reviewers’ assessments, the responses were also evaluated for word count and readability using the Flesch Reading Ease Score and Flesch-Kincaid Grade Level tools within Microsoft Word.

### Quantitative Analysis

Numerical responses on the Likert scale were compiled and displayed in box plots organized by question category corresponding to the 3 domains of accuracy, completeness, and overall provider satisfaction. The percentage of providers responding to the statements as either agree or strongly agree were calculated. Averages were calculated for word counts, Flesch Reading-Ease Score, and Flesch-Kincaid Grade Level. We used the Kruskal-Wallis test to evaluate the differences in responses between question categories and Wilcoxon rank-sum tests to evaluate the differences in responses between endocrine surgeons and endocrinologists.

### Qualitative Analysis

All free-text comments from both sets of responses were aggregated and organized by question and category for both endocrinologists and surgeons. An inductive qualitative content analysis was performed. The text was coded and subsequently organized into categories to identify common sentiments among the reviewers’ comments.

## Results

### Quantitative Analysis

Word count and reading difficulty statistics are shown in Table 1. The average word count of the ChatGPT responses was 300 words. The average Flesch Reading Ease Score and Flesch-Kincaid Grade Level were 25.8 and 14.6, corresponding with college graduate level and college level difficulty, respectively.

**Table 1.** Average word count and readability scores based on question category

Question category	Question number	Word count mean (SD)	Reading-ease mean (SD)	Grade level mean (SD)
Disease	1-3	204 (54.2)	27.2 (9.7)	15.3 (2.3)
Treatment	4-10	311 (86.9)	26.7 (9.0)	14.4 (1.7)
Follow-up	11-17	317 (47.4)	27.4 (6.6)	14.1 (1.4)
Psychosocial	18-19	292 (32.9)	18.9 (7.3)	15.6 (1.3)
Alternative	20	423 (17.0)	19.2 (1.8)	15.3 (0.4)
Total		300 (77.9)	25.8 (8.1)	14.6 (1.6)

**Figure 1.**
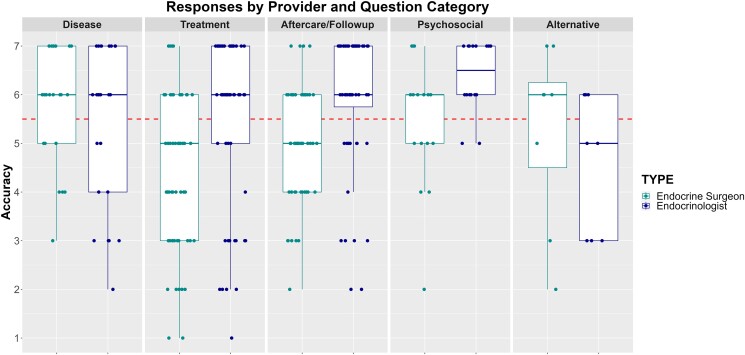
Likert-scale responses for accuracy. Scores above the dotted horizontal line indicate the provider “agreed” or “strongly agreed” that the response was accurate.

**Figure 2.**
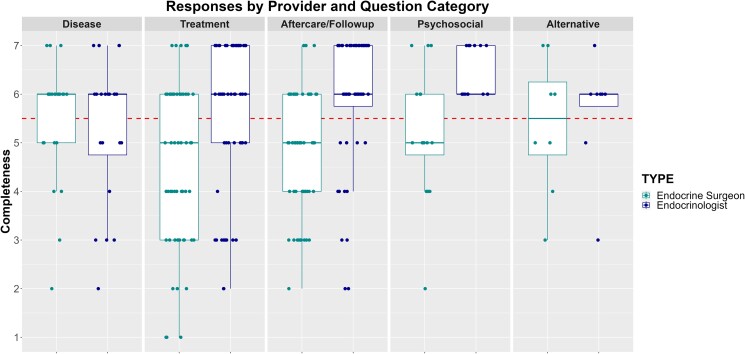
Likert-scale responses for completeness. Scores above the dotted horizontal line indicate the provider “agreed” or “strongly agreed” that the response was complete/comprehensive.

**Figure 3.**
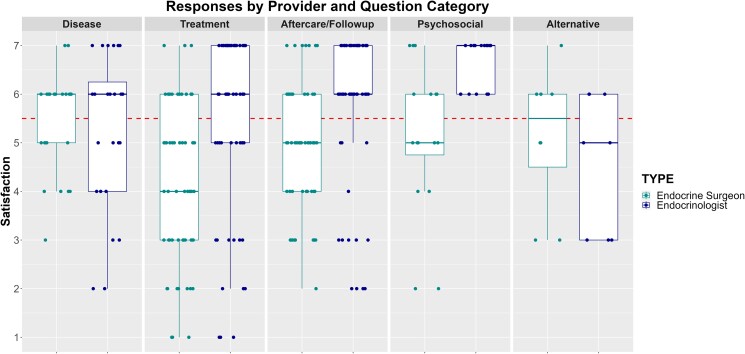
Likert-scale responses for satisfaction. Scores above the dotted horizontal line indicate the provider “agreed” or “strongly agreed” that they were satisfied with the response.

Table 2 reports the Likert-scale response data organized by question category and provider type. Figures 1-3 plot the scores for each question based on accuracy, completeness, and satisfaction respectively. Overall, 57%, 56%, and 52% of the responses agreed or strongly agreed that the AI-generated answers were accurate, complete, and satisfactory, respectively. Additionally, based on Kruskal-Wallis tests we found significant differences in responder ratings for accuracy (*P* = .03) and satisfaction (*P* = .02) based on question category, with treatment-related questions having the lowest ratings. The reviewers agreed or strongly agreed that treatment related questions were accurate 50% of the time and were satisfied only 45% of the time. Overall satisfaction was highest for the psychosocial-related questions, with reviewers agreeing or strongly agreeing that they were satisfied 72% of the time.

**Table 2.** Percent of responses answered “agree” or “strongly agree” based on question category

Category (number of questions)	Metric	Endocrine surgeons	Endocrinologists	Overall
Disease (3)	Accuracy	66.7%	62.5%	64.5%
	Completeness	66.7%	58.3%	62.5%
	Satisfaction	58.3%	54.2%	56.3%
Treatment (7)	Accuracy	30.4%	69.6%	50.0%
	Completeness	35.7%	62.5%	49.1%
	Satisfaction	26.8%	62.5%	44.6%
Aftercare/follow-up (7)	Accuracy	41.1%	75.0%	58.0%
	Completeness	33.9%	75.0%	54.5%
	Satisfaction	28.6%	76.8%	52.7%
Psychosocial (2)	Accuracy	56.3%	87.5%	71.9%
	Completeness	37.5%	100%	75.0%
	Satisfaction	43.8%	100%	71.9%
Alternative (1)	Accuracy	62.5%	37.5%	50.0%
	Completeness	50.0%	75.0%	62.5%
	Satisfaction	50.0%	37.5%	43.8%
Overall (20)	Accuracy	43.8%	70.6%	57.2%
	Completeness	40.6%	70.6%	55.6%
	Satisfaction	35.0%	68.8%	51.9%

Wilcox rank-sum tests showed that endocrine surgeons were more critical of the ChatGPT responses compared to the endocrinologists (*P* < .001). When asked if the response was accurate, endocrinologists agreed or strongly agreed 71% of the time, compared to 44% for endocrine surgeons. When asked if the response was complete/comprehensive, endocrinologists agreed or strongly agreed 71% of the time, compared to 41% for endocrine surgeons. When asked if they were satisfied with the response, endocrinologists agreed or strongly agreed 69% of the time, compared to 35% for endocrine surgeons. The evaluation of each individual question can be seen in Table 3.

**Table 3.** Number and percent of responses answered “agree” or “strongly agree” for individual questions

Category	Question	Metric	Endocrine surgeons	Endocrinologists	Overall
Disease	1. What is the thyroid?	Accuracy	75%	100%	88%
		Completeness	63%	50%	56%
		Satisfaction	63%	88%	75%
	2. What causes thyroid cancer?	Accuracy	63%	38%	50%
		Completeness	75%	88%	81%
		Satisfaction	63%	38%	50%
	3. Is thyroid cancer hereditary?	Accuracy	63%	50%	56%
		Completeness	63%	38%	50%
		Satisfaction	50%	38%	44%
Treatment	4. How is thyroid cancer treated?	Accuracy	63%	100%	81%
		Completeness	75%	63%	69%
		Satisfaction	50%	75%	63%
	5. What are the risks of thyroid surgery?	Accuracy	50%	63%	56%
		Completeness	38%	50%	44%
		Satisfaction	25%	50%	38%
	6. What is the recovery like after thyroid surgery?	Accuracy	0%	75%	38%
		Completeness	0%	75%	38%
		Satisfaction	0%	75%	38%
	7. How much pain will I be in after thyroid surgery?	Accuracy	13%	100%	56%
		Completeness	25%	88%	56%
		Satisfaction	13%	88%	50%
	8. What are the risks of radioactive iodine treatment for thyroid cancer?	Accuracy	25%	25%	25%
		Completeness	38%	63%	50%
		Satisfaction	38%	38%	38%
	9. How do I isolate after radioactive iodine treatment?	Accuracy	50%	88%	69%
		Completeness	50%	50%	50%
		Satisfaction	50%	63%	56%
	10. Will radioactive iodine affect fertility?	Accuracy	13%	38%	25%
		Completeness	25%	50%	38%
		Satisfaction	13%	50%	31%
Follow-up	11. What are the long-term effects of thyroid cancer treatment?	Accuracy	25%	63%	44%
		Completeness	25%	88%	56%
		Satisfaction	13%	88%	50%
	12. How do I treat low calcium after thyroid surgery?	Accuracy	25%	100%	63%
		Completeness	25%	75%	50%
		Satisfaction	25%	88%	56%
	13. How do I know if my thyroid cancer has come back?	Accuracy	50%	75%	63%
		Completeness	13%	75%	44%
		Satisfaction	25%	88%	56%
	14. If my thyroid cancer comes back, what treatment options are there?	Accuracy	63%	88%	75%
		Completeness	38%	75%	56%
		Satisfaction	38%	75%	56%
	15. Will I gain weight after thyroid surgery?	Accuracy	50%	100%	75%
		Completeness	50%	100%	75%
		Satisfaction	38%	100%	69%
	16. What kind of exercises should I do after my thyroid surgery?	Accuracy	50%	63%	56%
		Completeness	63%	63%	63%
		Satisfaction	50%	63%	56%
	17. What is the best diet for thyroid cancer patients?	Accuracy	25%	38%	31%
		Completeness	25%	50%	38%
		Satisfaction	13%	38%	25%
Psychosocial	18. What are the best resources for thyroid cancer patients?	Accuracy	50%	75%	63%
		Completeness	25%	100%	63%
		Satisfaction	38%	100%	69%
	19. Are there support groups for thyroid cancer patients?	Accuracy	63%	100%	81%
		Completeness	50%	100%	75%
		Satisfaction	50%	100%	75%
Alternative	20. Are there any alternative or natural treatments for thyroid cancer?	Accuracy	63%	38%	50%
		Completeness	50%	75%	63%
		Satisfaction	50%	38%	44%

### Qualitative Analysis

A total of 198 free text comments were recorded between both sets of ChatGPT responses (106 from endocrinologists, 92 from surgeons). Although there were a mix of both positive and negative comments, the majority of comments were critical in nature.

#### Disease-related questions

The majority of the comments were supportive in regard to the basic question of “what is the thyroid?” The reviewers felt the responses were adequate, although several desired more or different information be included. While most of the surgeons’ comments were supportive in response to “what causes thyroid cancer?”, the comments from endocrinologists were largely critical. Multiple endocrinologists and surgeons felt the responses overemphasized the effect of diet and iodine intake on risk of cancer. Additionally, the responses included presence of a goiter or thyroiditis as risk factors for thyroid cancer, which multiple reviewers felt might cause unnecessary anxiety for patients. Finally, in response to “Is thyroid cancer hereditary?”, there were several positive comments, though reviewers felt the answer was incomplete as the list of genetic syndromes included in the response was not very comprehensive. Again, 1 of the responses mentioned diet and lifestyle contributing to thyroid cancer risk, which several of the reviewers disagreed with.

#### Treatment-related questions

Comments regarding the overview of thyroid cancer treatment were fairly positive, with several reviewers being pleased or impressed with the responses. One of the ChatGPT responses included active surveillance and the other did not, which the reviewers preferred included in the list of treatment options. For responses specifically related to risks of thyroid surgery, the reviewers’ comments were more critical, citing a lack of emphasis on parathyroid injury, inappropriate information such as risk of thyroid storm, and not including rates of risk. The reviewers also felt the information on hospital length of stay and need for prescription pain medication was inaccurate. Finally, the comments highlighted multiple concerns about the responses to radioactive iodine (RAI)-related questions. The chief concern was the overemphasis of RAI's effect on fertility. The responses from ChatGPT stated that RAI could cause temporary or permanent infertility in both males and females, damage to reproductive organs, and permanent menopause-like symptoms. The reviewers felt this would cause significant anxiety for patients, which is largely unnecessary as the information is inaccurate and not evidence-based. Additional comments highlighted lack of completeness, inaccuracies regarding the risk of secondary malignancies, and generally inappropriate information (for example, ChatGPT seemingly mistook the risks of RAI therapy for hyperthyroidism with the risks of RAI for thyroid cancer therapy).

#### **Aftercare/long-term follow-u**p **questions**

The comments regarding the general question about long-term effects of thyroid cancer treatment are mixed, but mostly critical in nature. The comments suggest the information is too generic or vague, and some of the information included is once again inappropriate (example: external beam radiation leading to hypothyroidism, despite the fact the patient would already be hypothyroid following thyroidectomy). In regard to treatment of hypocalcemia, ChatGPT included recombinant PTH as a potential option, which the reviewers pointed out was not available in the United States at the time of this study following a safety-related recall in 2019. In terms of monitoring for cancer recurrence, 1 of the ChatGPT responses placed more emphasis on regular follow-up with biochemical and imaging surveillance, whereas the other response seemed to put a larger emphasis on signs and symptoms. The reviewers felt some of the signs and symptoms were overemphasized, such as fatigue, weakness, or pain, which are not typically signs of recurrence. The responses regarding treatment options for recurrent thyroid cancer were fairly well received by the reviewers, though several reviewers highlighted the lack of active surveillance as an option in one of the responses. Suppression of TSH was also missing from the responses. In regard to exercise after surgery, reviewers commented that the information was overall acceptable, but missed 1 of the most commonly cited recommendation from surgeons; no heavy lifting in the immediate postoperative period. Finally, the reviewer comments were mixed on the question regarding diet for patients with thyroid cancer. There was a large emphasis on dietary iodine intake, which the reviewers felt was inappropriate and not evidence-based. That said, several reviewers appreciated the fact that the answer from ChatGPT included a disclaimer and recommended discussion with a health care provider.

#### Psychosocial questions

Most of the reviewers felt the list of resources provided by ChatGPT were complete and appropriate, although the surgeons would have liked to see the American Association of Endocrine Surgeons website included as a resource, particularly as a way for patients to find experienced thyroid surgeons.

#### Alternative/complementary medicine questions

In regard to the question on alternative or complementary medicine, the reviewers cited a lack of evidence to support much of what was included in the responses from ChatGPT. However, the reviewers were also appreciative of the multiple disclaimers made in the responses, and that the responses emphasized that alternative medicine should not replace conventional treatment.

## Discussion

As ChatGPT continues to serve as a highly utilized and evolving resource by the general public, an increasing number of articles have explored its capability to provide health-related information for patients. Although the results have been mixed, the general conclusion is that large language model AI programs such as ChatGPT hold promise as a means to answer patient questions. While most studies have utilized a quantitative approach to assess this, our study also incorporated a qualitative analysis to gain additional insight into some of the strengths and shortcomings of ChatGPT in answering thyroid cancer-related questions.

When evaluating the data from the Likert-scale ratings, we opted to organize the data in terms of percentage of times a provider agreed or strongly agreed that ChatGPT was accurate, complete, or satisfactory. We felt that if a response scored less than “agree,” we would not feel comfortable if that response was provided to our patients. Surprisingly, the responses only met this threshold for the reviewers just over 50% of the time. This indicates that many of the responses would not be universally considered acceptable.

The comments from reviewers provided a great deal of insight. Although some of the comments were positive, the majority of the comments were critical in nature. Both endocrinologists and surgeons appreciated when ChatGPT included a disclaimer about the information provided and suggested discussing with a health care professional. Many of the responses from ChatGPT did include a disclaimer; however, it is unclear how often patients may ignore the disclaimer and accept the information as-is without actually discussing with their provider. There is concern that a growing number of patients place more trust in internet resources, AI, and social media than health care providers. A public opinion survey conducted in 2023 polled 2000 adults in the United States regarding health information-seeking behavior. More respondents indicated they turn to internet resources for health information than they do their doctor (53% vs 44%) [29]. Fifty-three percent of the respondents indicated they would trust AI to recommend treatment plans. These sentiments may make it more challenging for health care providers to correct misinformation that patients have obtained from internet resources.

Although we did not aim to evaluate ChatGPT's consistency in its responses, we did ask the same set of questions twice on different days. There were only a few notable inconsistencies that emerged in the reviewer comments. When asked about treatment options, 1 response included active surveillance, whereas the other did not. A more noticeable and concerning difference occurred when asked about detecting recurrence. One response focused heavily on routine health care visits, laboratory testing, and imaging as the primary means of detecting recurrence, whereas the other response focused on symptoms, some of which were inappropriate or inaccurate (for example, the presence of fatigue or pain). This could lead the patient to seek additional tests that may not be medically necessary, such as more frequent ultrasound surveillance.

A major point of concern among the reviewers was the information provided on risks of RAI. It seemed this was in part due to ChatGPT confusing the effects of RAI as treatment for hyperthyroidism as opposed to thyroid cancer, as well as ChatGPT confusing RAI with other forms of radiation therapy for cancer. Particularly notable were the statements regarding RAI and fertility. ChatGPT suggested RAI could cause temporary or permanent infertility in both males and females, damage reproductive organs, affect hormone levels necessary for fertility, and cause temporary or permanent menopause-like symptoms. Although these are all potential complications from other forms of radiation therapy, this is not necessarily true for RAI delivered at typical doses. Although the data on RAI and fertility are not robust, current evidence suggests that there is no long-term risk of infertility. In women, RAI has been associated with temporary oligomenorrhea or amenorrhea, a slightly earlier age of menopause, mild decrease in anti-mullerian hormone, and increased rates of miscarriage within the first year after therapy [30-32]. Despite some of these observations, birth rates appear similar between patients who do and do not receive RAI [30, 31]. There are fewer data on male fertility; however, current data suggest a decrease in sperm count within 3 months of therapy, with return to normal by 12 months [33]. The most common counseling that patients receive about the effect on fertility is to delay conception for 6 to 12 months given the observed increase in miscarriage within the first year. This was not included in ChatGPT's response. This is important given that thyroid cancer is the most common cancer among women aged 20-30, and the second most common cancer in women from ages 30 to 39 years [34]. Since many thyroid cancer patients are in their peak reproductive years, fertility concerns are of major importance. In the aforementioned survey regarding individuals’ trust in internet resources for health information, 40% of respondents trusted AI with questions related to fertility. The answers provided by ChatGPT related to RAI and fertility risk are likely to increase worry and anxiety in young patients with thyroid cancer.

With advancements in medical knowledge and practice changing ever more rapidly, programs such as ChatGPT may include outdated information in its responses to inquiries. The reviewers felt this may have been the case with some of the responses to our thyroid cancer-related questions. When asked about recovery from surgery, ChatGPT mentioned a 1- to 2-day hospital stay. The surgeons in general felt this was overestimating the length of stay and wanted to see outpatient same-day surgery included in the response as well. When commenting on the need for postoperative pain medication, ChatGPT suggested pain medication would be prescribed. Although the responses from ChatGPT were somewhat vague, the surgeons felt the responses implied the patient would need prescription pain medication. Research has shown that most patients undergoing thyroid surgery need very little, if any, opioid pain medication, and thyroid surgeons rarely prescribe them routinely, making the response from ChatGPT somewhat misleading [35]. Finally, ChatGPT include recombinant PTH as a treatment option for hypoparathyroidism. Although this can sometimes be obtained off-label in the United States utilizing medications marketed for osteoporosis, the Food and Drug Administration-approved version for hypoparathyroidism was recalled in 2019 and has not been reintroduced. Outdated information likely contributed to the inaccuracy of this answer from ChatGPT. Finally, there was a general theme that diet and lifestyle, particularly iodine intake, play an important role in thyroid cancer development and aftercare. There are minimal data on iodine intake and risk of thyroid cancer, and even less evidence on other dietary and lifestyle factors. It is unclear why ChatGPT seemed to emphasize these factors but given the numerous non-evidence-based websites and books for thyroid support and advice, one can speculate that these contributed in some way to the responses.

Several studies have evaluated the readability of ChatGPT responses using standardized readability formulas such as the Flesch Reading-Ease Score, Flesch Kincaid Grade Level, Gunning Fog index, and Simple Measure of Gobbledygook scores. Our results were in concordance with other studies, demonstrating ChatGPT's responses are typically above the suggested 8th-grade reading level for patient material [26, 36, 37]. Although one could ask ChatGPT to provide the responses at an 8^th^-grade reading level, this was not completely effective in a prior study [26]. The Agency for Healthcare Research and Quality has pointed out limitations with using these formulas to determine readability and understanding. These formulas primarily take into account word length and sentence length and operate under the assumption that longer words and sentences are more difficult to understand. Medical vocabulary contains many words that are long, but may still be familiar to readers, and thus should not influence the reader's understanding. Along these same lines, a well-crafted sentence may be very understandable to the reader, even if it is long. Additionally, how information is organized can play a significant role in enhancing the reader's understanding. When calculating these readability scores, one must remove extra punctuation and formatting so it does not influence the score. This poses a problem when evaluating ChatGPT's responses because many responses attempt to organize the material in an understandable fashion utilizing headings and lists. The reviewers in our study rarely commented about concerns regarding readability from a patient perspective. Although these readability scores have largely been the preferred method to evaluate patient education materials, different approaches should be developed to address some of the limitations specifically pertaining to health information for patients.

Our study has several limitations. Only 20 unique questions were used to evaluate ChatGPT's responses. Because patients can ask any number of questions, which may or may not be worded appropriately or grammatically correctly, it is unclear how ChatGPT would perform in the hands of patients. ChatGPT's responses can be influenced by previous responses within a conversation or thread. We deliberately used separate conversations for each question posed to ChatGPT. That said, patients are unlikely to do this, and it is unclear how the order of questions would influence the results. Although our study included 8 reviewers with expertise in thyroid cancer, this is still a limited sample size, and our scoring methodology has not been validated. Reviewers were not blinded to the fact that these answers were generated by AI, potentially introducing bias. Finally, we utilized ChatGPT 3.5 for our study given it is free to the public. It is unclear if the newer version, ChatGPT 4, would have provided different responses.

## Conclusion

The sub-par scores as well as critical comments from eight thyroid cancer specialists suggest ChatGPT is not accurate or reliable enough at this time for unsupervised use as a patient information tool for thyroid cancer. Alternative approaches are needed to optimize AI for patient information, such as use of retrieval-augmented generation focusing on authoritative evidence-based literature and thyroid cancer resources.

## Data Availability

Original data generated and analyzed during this study are included in this published article or in the data repositories listed in References.

## Abbreviations

AI: artificial intelligence
DTC: differentiated thyroid cancer
RAI: radioactive iodine
